# High Proton Conducting Polymer Blend Electrolytes Based on Chitosan:Dextran with Constant Specific Capacitance and Energy Density

**DOI:** 10.3390/biom9070267

**Published:** 2019-07-09

**Authors:** Shujahadeen B. Aziz, M. H. Hamsan, Wrya O. Karim, M. F. Z. Kadir, M. A. Brza, Omed Gh. Abdullah

**Affiliations:** 1 Advanced Polymeric Materials Research Lab., Department of Physics, College of Science, University of Sulaimani, Qlyasan Street, Sulaimani 46001, Kurdistan Regional Government, Iraq; 2 Komar Research Center (KRC), Komar University of Science and Technology, Sulaimani 46001, Kurdistan Regional Government, Iraq; 3 Centre for Foundation Studies in Science, University of Malaya, Kuala Lumpur 50603, Malaysia; 4 Department of Chemistry, College of Science, University of Sulaimani, Qlyasan Street, Sulaimani 46001, Kurdistan Regional Government, Iraq; 5 Faculty of Engineering, International Islamic University of Malaysia, Kuala Lumpur, Gombak 53100, Malaysia

**Keywords:** biopolymer, polymer blend electrolyte, XRD and FTIR analysis, impedance study, morphology study, TNM and LSV study, CV plot, EDLC study

## Abstract

Polymer blend electrolytes based on chitosan: dextran (CS:Dext) incorporated with various amounts of ammonium fluoride (NH_4_F) with constant specific capacitance (12.4 F/g) and energy density over 100 cycles were prepared using a solution cast technique. The blend electrolyte samples exhibit broader amorphous humps in X-ray diffraction (XRD) spectra compared to pure CS:Dext film. The Fourier transform infrared (FTIR) study indicates the complex formation of the added ammonium salt with the polymer blend functional groups through the shifting and decrease in the intensity of FTIR bands. The impedance plots were used to determine the conductivity of the samples. The field emission scanning electron microscopy (FESEM) images support the conductivity behavior of the samples. The impedance plots were applied in the determination of the conductivity of the samples in which the relatively highest conductivity was gained to be 1 × 10^−3^ S/cm. The transference number measurement (TNM) of the conducting electrolyte was 0.88, which portrays the dominancy of ion in the conduction process. Linear sweep voltammetry (LSV) verified the chemical stability and showed it to be 1.7 V and an effective electrical double layer capacitor (EDLC) that is applicable in electrochemical devices. The performance of the EDLC cell was examined using both cyclic voltammetry and constant current charge–discharge techniques at ambient temperature. The semi-rectangular shape of the cyclic voltammetry (CV) plot and no redox peak was observed. The charge-discharge process of the fabricated EDLC is durable over 100 cycles with an equivalent circuit resistance and power density of 194.5 Ω and 428 W/kg, respectively. Two main outcomes, the specific capacitance and energy densities of 12.4 Farad/g and 1.4 Wh/kg, respectively, are almost constant over 100 cycles.

## 1. Introduction

The modern lifestyle involves an increasing demand for the latest electronic devices, which indirectly increases electrical waste in the natural environment. Orlins et al. [1] documented the second highest contributor of electrical waste of electronic devices, which is mobile phone waste. An effective way to reduce this kind of electronic waste is having more biodegradable materials in the devices. Thereby, biodegradable biopolymers are a well-defined alternative to the non-biodegradable and synthetic ones [2]. Several natural biopolymer resources have been studied as the electrolyte of energy devices by numerous researchers, including starch, dextran, cellulose, chitosan, carrageenan and algae [3,4,5]. Among diverse renewable natural polymers, chitosan is one of the major commercially essential biocompatible polymers from a green or biomedical viewpoint [4]. These kinds of biopolymers exhibit plausible film forming characteristics, for example, cheapness, ease of preparation and compatibility with various solvents. If one looks at these biopolymer backbones, it is seen that there are different kinds of oxygen containing functional groups enriched in electron lone pairs. The conduction mechanism comes from the ions of the salt that have the ability to make a dative bond with these functional groups [6]. Dextran is a non-toxic biopolymer produced by a culturation process of *Leuconostocmesenteroides* bacteria in an environment of sucrose. During this process, an enzyme called dextransucrase is produced, which is then converted to dextran. The polymer chain of dextran consists of 1,6-α-D-glucopyranosidic linkages with oxygen containing functional groups (OH and C-O-C). There are various applications of dextran, for example, blood substitutes, plasma expanders and bone curing [7].

On the one hand, one issue related to the study of dextran film is brittleness. On the other hand, to improve the flexibility of dextran film, it is necessary to involve plasticizer, filler, salt and polymer blending [8]. For example, a successful methodology for improving the conductivity of polymer electrolyte is the synthesis of a polymer blend. These kinds of polymer are formed by complexation sites for ionic conduction [9]. In a comparison reported by Hamsan et al. [10], a methylcellulose-starch blend incorporated by ammonium nitrate (NH_4_NO_3_) has provided higher ionic conductivity than methylcellulose-NH_4_NO_3_ [11] and starch-NH_4_NO_3_ [12]. A deep understanding of the mechanism of the ionic conductivity of these polymers is related to both the amorphous region and the flexible polymer chain. To lower both glass transition temperature and the degree of crystallinity, a proper methodology is to blend dextran and chitosan, as explored in previous work [13]. The common electrode separators, H^+^ ion (proton) based polymer electrolytes, have been used in electrochemical device applications [14,15]. Ammonium salt and inorganic acids are H^+^ ion providers, for instance phosphoric acid (H_3_PO_4_) and sulfuric acid (H_2_SO_4_) based polymer electrolyte, which are characterized by experiencing chemical degradation and poor mechanical integrity [16,17]. Therefore, ammonium salts are the H^+^ ion provider of choice in polymer electrolytes because of their compatibility, high ionic conductivity and thermal stability [18,19]. Another alternative for conventional batteries is the electrochemical double-layer capacitor (EDLC), where the energy storage mechanism is charge adsorption rather than intercalate/deintercalate process [20]. Various active materials have been employed for EDLC electrodes such as graphite [21], carbon aerogel [22], carbon nanotubes [23] and activated carbon [24]. Among them, activated carbon is recognized as having are latively large surface area, excellent chemical durability and high electronic conductivity [25]. 

In this current work, a chitosan-dextran blend host has been incorporated with different amounts of ammonium fluoride (NH_4_F). The structural, morphological and electrical properties of the electrolyte have been explored using X-ray diffraction (XRD), Fourier transform infrared (FTIR) spectroscopy, field emission scanning electron microscopy (FESEM) and electrical impedance spectroscopy (EIS). The role of the highest conducting chitosan-dextran-NH_4_F film is as an electrode separator in the fabricated EDLC cell.

## 2. Experimental Method

### 2.1. Materials and Sample Preparation

High molecular weight chitosan (CS) (average molecular weight 310,000–375,000) and Dextran powder (average molecular weight 35,000–45,000) materials were used as the raw materials (Sigma-Aldrich, Warrington, PA, USA). For the fabrication of the polymer blending based on CS:Dextran, 60 wt.% chitosan and 40 wt.% dextran were dissolved separately in 50 mL of 1% acetic acid at room temperature for 90 min. Subsequently, these solutions were then mixed and stirred for 3 h to gain a homogeneous blending solution. For the blended solution of CS:Dextran, various amounts of NH_4_F ranging from 10 to 40 wt.% in steps of 10 were added separately with continuous stirring to prepare CS:Dextran:NH_4_F polymer blend electrolytes. The samples were coded as CSDX1, CSDX2, CSDX3, and CSDX4 for CS: Dextran and incorporated with 10, 20, 30, and 40 wt.% of NH_4_F, respectively. After casting in different Petri dishes, the solutions were left to dry at room temperature for films to form. The films were transferred into a desiccator for further drying, which produced solvent-free films.

### 2.2. TNM Technique

A V&A Instrument DP3003 (SHANGHAI YIHUA V&A INSTRUMENT CO.LTD, Shanghai, China) digital DC power supply was used to analyze ionic (*t_i_*) and electronic (*t_e_*) transference number. The highest conducting electrolyte was placed in a Teflon holder with identical stainless steel electrodes. The cell was subjected to0.80 V at room temperature. *t_i_* was identified using the following equation [26]:

(1) $$t_{i}=\frac{I_{i}-I_{ss}}{I_{i}}$$

(2) $$t_{i}=1-t_{e}$$

Here, *I_ss_* and *I_i_* are the steady state and initial current, respectively.

### 2.3. LSV

To study the electrochemical stability of the electrolyte, linear sweep voltammetry (LSV) was done via a Digi-IVY DY2300 potentiostat. The electrolyte was sandwiched between two stainless steel electrodes of a Teflon holder with an applied scan rate of 50 mV s^−1^.

### 2.4. EDLC

Carbon black of 0.25 g was mixed with 3.25 g activated carbon using a planetary ball miller prior to the mixture being poured into a solution of 15 mL N-methyl pyrrolidone (NMP) and 0.50 g of polyvinylidene fluoride (PVdF). A dark black solution appeared upon dissolution. An aluminum foil was cleaned with acetone and flattened on a glass surface. The solution was poured and coated on the foil using a doctor blade. The electrodes were left for a period in the oven to dry at 60 °C. Then, the dried electrodes were kept in a desiccator for further drying. An electrode of 2.01 cm^2^ was made in the shape of a small circle. The electrolyte with the highest conductivity was placed between two activated carbon electrodes and packed in a CR2032 coin cell shape. The properties of the EDLC were verified using cyclic voltammetry (CV) analysis at a sweep rate of 100 mV s^−1^. The charge-discharge profiles of the EDLC were explored using aNeware battery cycler with a current density of 0.2 mA cm^−2^. The specific capacitance (*C_sp_*) of the EDLC

(3) $$C_{sp}=\frac{i}{gm}$$

where *i* is the operating current, *g* is the gradient of discharge curve and *m* is the active material’s mass. Other parameters of the EDLC, such as equivalent series resistance (*R_es_*), energy density (*E*) and power density (*P*), can be expressed as [3]:

(4) $$R_{es}=\frac{V_{drop}}{i}$$

(5) $$E=\frac{C_{s}V}{2}$$

(6) $$P=\frac{V^{2}}{4mR_{es}}$$

Here, *V_drop_* stands for voltage drop before the discharging process and *V* is the voltage applied.

## 3. Results and Discussion

### 3.1. Structural (XRD and FTIR) Analysis

**Figure 1:**
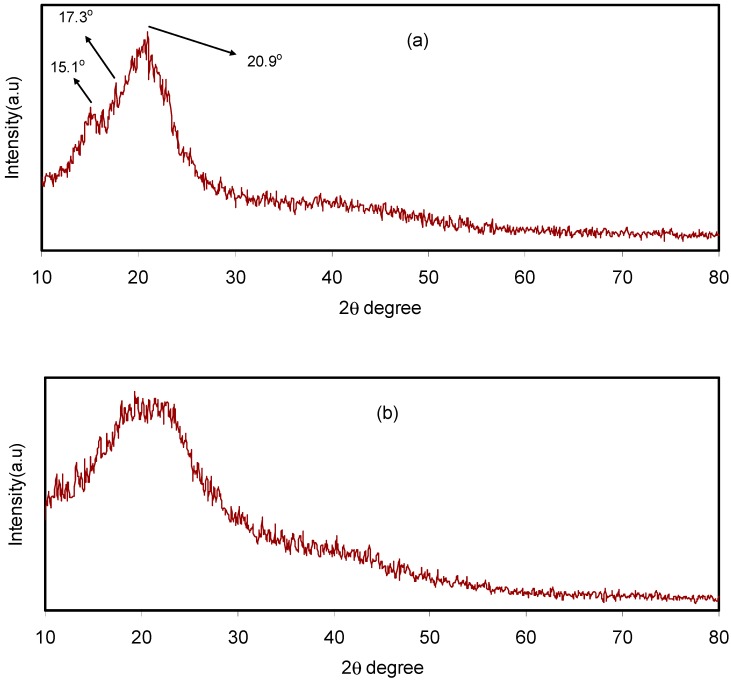
X-ray diffraction (XRD) pattern for (**a**) pure chitosan (CS) and (**b**) CS:Dex blend at ambient temperature.

Pure CS film, CS:dextran blend and CS:dextran:LiClO_4_ blend electrolyte complexes were subjected to XRD at room temperature. The CS has crystalline peaks at the 2θ values of 15.1°, 17.7° and 20.9° [27,28] (Figure 1a), whereas dextran has two broad peaks at 2θ values of 18° and 23° [29], as demonstrated in previous work. In the current work, two broad peaks and no crystalline peaks appeared in the XRD pattern of the CS:dextran blend film (Figure 1b). It is interesting that, as indicated by the broad peaks, the CS:dextran blend is not as crystalline as raw chitosan and its structure is nearly amorphous [30,31]. According to previous studies, the amorphous nature of polymer electrolyte is correlated with a broad diffraction peak [32,33].

**Figure 2:**
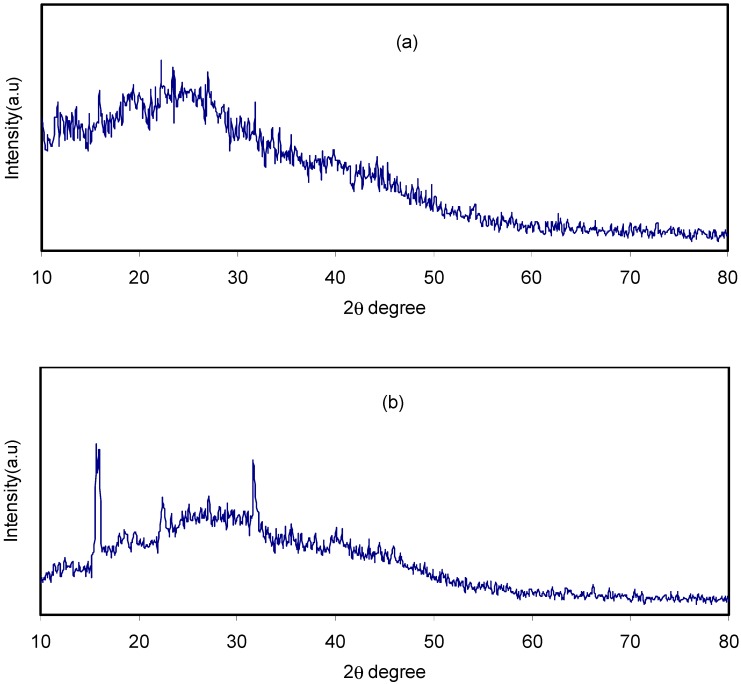
XRD pattern for (**a**) CSDX2 and (**b**) CSDX2 blend electrolyte samples at ambient temperature.

Meanwhile, this study has observed that, when NH_4_F salt was added, the CS:dextran hallow displayed intensity reduction and its broad nature was enhanced as depicted Figure 2a,b. Such observations validate the amorphous nature of polymer electrolytes, which promotes better conductivity by improving ionic diffusivity. Furthermore, the NH_4_F salt undergoes full dissociation in the polymer blend matrix as for the CS:dextran blend polymer electrolyte, there is no peak related to pure NH_4_F. The elimination of hydrogen bonding between the polymer chains is a likely reason for the broadening at 40 wt.% NH_4_F salt and the reduction in intensity, signifying the prevalence of the amorphous phase within the sample [34,35]. Moreover, the polymer blend electrolyte displays few crystalline peaks. A sort of a long-range order established by the existence of ion multiples may explain such new peaks [36]. By contrast, Sanders et al. claim that the formation of polymer-salt complexes and not pure salt is the reason for the new peaks [37]. Additionally, there are reports of these peaks occurring at lower 2θ degrees in chitosan-based electrolyte [38].

**Figure 3:**
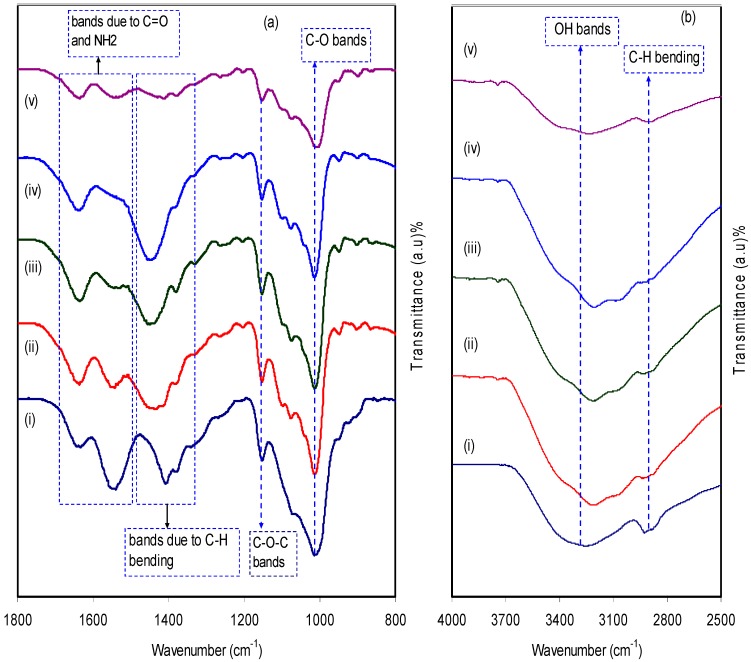
Fourier transform infrared (FTIR) spectra of (i) CS:Dextran (pure blend film), (ii) CSDX 1, (iii) CSDX 2, (iv) CSDX 3, and (v) CSDX 4 in the region (**a**) 800 cm^−1^ to 1800 cm^−1^, and (**b**) 2500 cm^−1^ to 4000 cm^−1^.

Figure 3a,b illustrates the FTIR spectra of both pure CS:Dextran and the blend electrolyte films at two distinct regions separately. Fourier transform infrared (FTIR) spectroscopy was applied in an attempt toverify the formation complexes in polymer blend electrolytes. Also, the technique provides insight into intermolecular interaction via analysis of FTIR spectra on the basis of the stretching or bending vibrations of specific bonds. Thereby, a band peak centered at 2906 cm^−1^ can be ascribed to C–H stretching in dextran [39,40], because this band was absent in the FTIR spectra of pure chitosan [41] and almost disappeared at high salt concentration. The hydroxyl band in CS:Dextran which peaked at 3253 cm^−1^ showed a shift on the inclusion of various amounts of the ammonium fluoride salt and their intensity lowered significantly. This indicates the dative bond formation between cations with the oxygen atoms of the host polymer blends [29]. Another two peaks at 1007 cm^−1^ and a small one at 1074 cm^−1^ suggest the existence of C-O bonds [41]. As the concentration of ammonium fluoride salt increased, these peaks became wider and lowered in intensity. The bending mode of C–H usually peaked at 1450 cm^−1^ whereas a broad band which commences at 1149 cm^−1^, indicating asymmetrical –C–O–C– stretching of the ring [40]. Two peaks appeared at 1632 cm^−1^ and 1544 cm^−1^ and correlated with carboxamide (O=C–NHR) and amine (NH_2_) bonds, respectively [41]. It is interesting that observation of a shift in the carboxamide (O=C–NHR) and amine (NH_2_) bonds strongly emphasizes a complexation between chitosan:Dextran and the dopant salt. Clearly, this cation salt to nitrogen and oxygen atom attachments reduces the vibration intensity of the N–H or O=C–NHR bonds as a consequence of obtaining the higher molecular mass resulting from cation binding and eventually resulted in both shifting and lowering in peak intensity [42]. An incredibly interesting observation is the incorporation of NH_4_F salt into the CS:Dextran composite, resulting in a remarkable change in the intensity of the bands. It is apparent that the change in intensity of these bands is strongly correlated with the alterations in the macromolecular order. In fact, these bands of the resulting system may result from the extent of the ordered structures [43]. All these were emphasized by the XRD results as presented in Figure 1.

### 3.2. Impedance and Morphology Study

**Figure 4:**
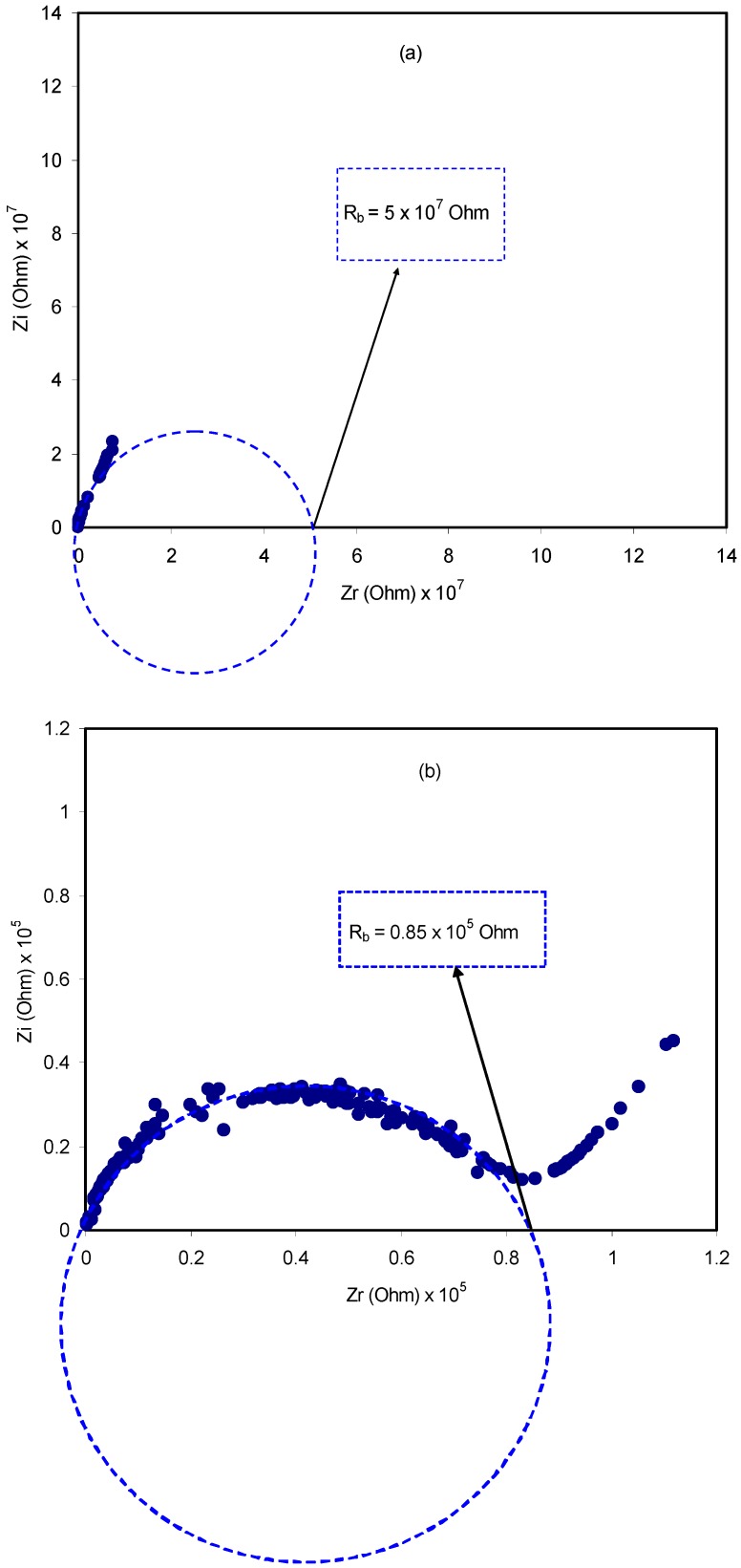

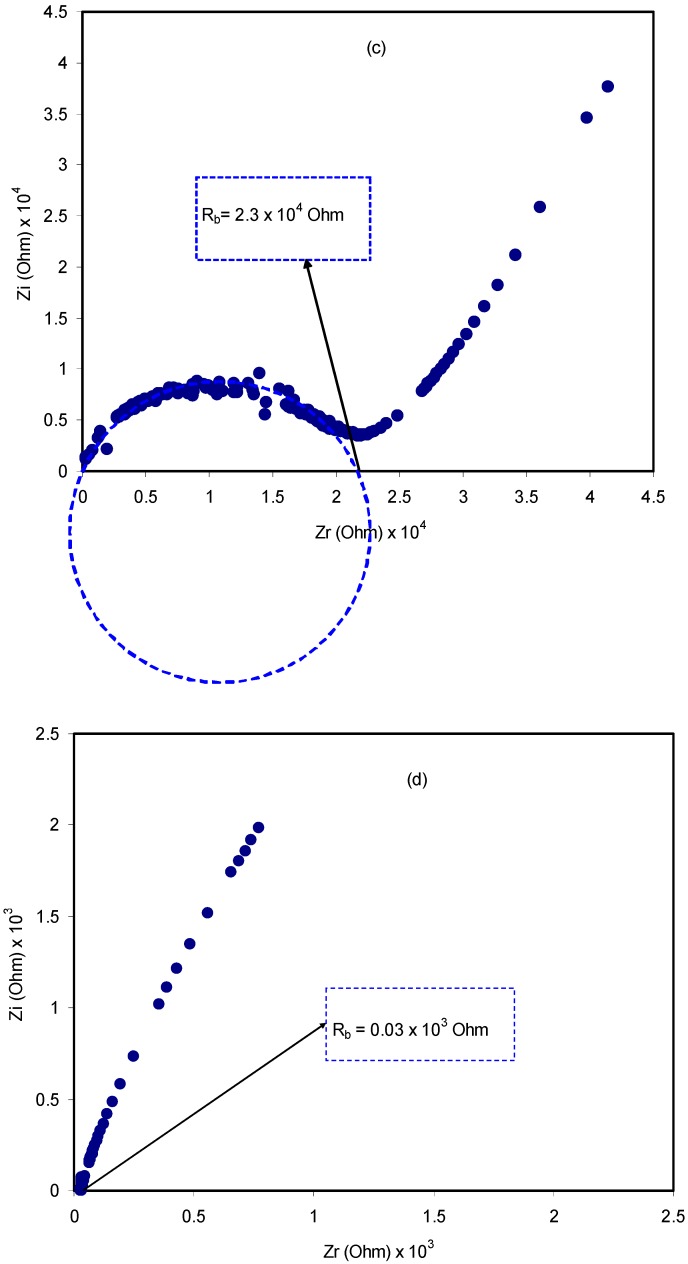

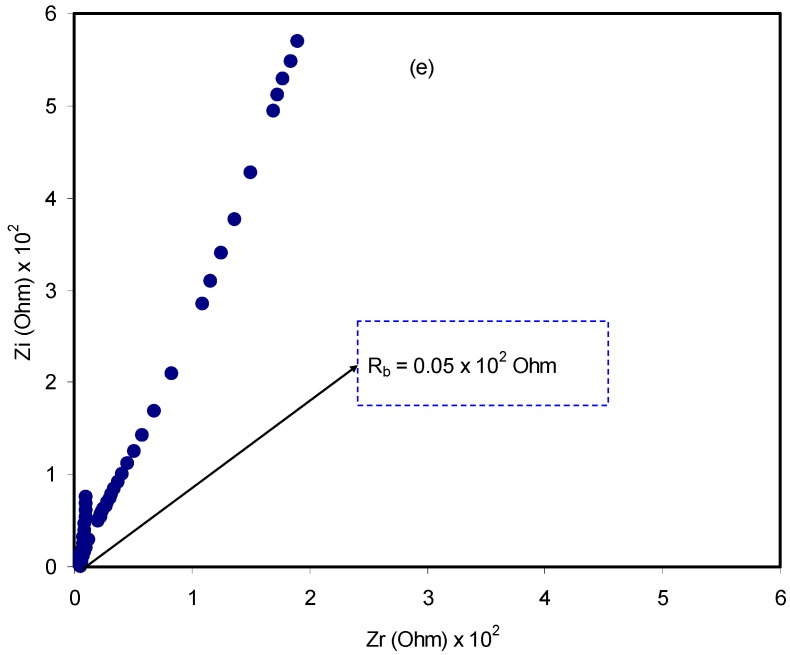
Impedance plots for (**a**) pure CS:Dex film, (**b**) CSDX1, (**c**) CSDX2, (**d**) CSDX3, and (**e**) CSDX4 blend electrolyte films.

A relatively novel and robust approach is impedance spectroscopy, which facilitates the exploration of a number of electrical properties of electrolyte materials and their interfacial regions with electronically conducting electrodes [44]. The impedance of pure CS:dextran and blend electrolytes at room temperature is shown in Figure 4a–e. In the case of a pure composite system, it is likely to notice only a semicircle. One the one hand, at a high-frequency a semicircle and a tail are seen at 10 wt.% and 20 wt.% NH_4_F. On the other hand, the response of the transport of ions across the bulk of the electrolyte is located in the high-frequency region and ion accumulation at the electrode/electrolyte interfacial region is the cause of the low-frequency tail, resulting in the formation of double-layer capacitance [45,46,47]. Thus, ion accumulation on both sides of the electrolyte membrane will produce electrical double layer capacitances. The results clearly indicated that, with increasing salt concentration, from 10 to 40 wt.%, the semicircle decreased and the contribution of tail regions increased and therefore the resistance decreased due to the large amount of carrier density. The semicircle at a high-frequency is equivalent to the parallel connection of the bulk resistance (R_b_) and bulk capacitance model for the polymer electrolytes [48,49,50,51]. In the Nyquist plot, the semicircle disappears at 30 wt.% and 40 wt.% of the NH4F salt (see Figure 4d,e), suggesting that only the resistive component of the polymer prevails [49]. The *R_b_* value is determined by these points where the semicircle (at low salt concentration) or the tail (at high salt concentration) intersects the real axis (*Z_r_*). Based on the *R_b_* value and the sample dimensions, the equation below has been taken into consideration in the determination of the sample conductivity:

(7) $$\sigma_{dc}=\left(\frac{1}{R_{b}}\right)\times \left(\frac{t}{A}\right)$$

where the polymer electrolyte film thickness and the film surface area are denoted by *t* and *A,* respectively. It is worth-mentioning that a unique spike region is seen at 30 wt.% and 40 wt.% NH_4_F, suggesting that ion diffusion is the only mechanism of ion transport [52]. To pinpoint, a maximum conductivity of 1 × 10^−3^ S/cm is displayed by CS:dextran doped with 40 wt.% NH_4_F at ambient temperature. Obviously, the addition of NH_4_F in various concentrations has significantly improved the DC ionic conductivity of CS:dextran-based electrolytes that are tabulated in Table 1. The rise in both charge carrier concentration and mobility would enhance DC conductivity, which is generally expressed as follows at room temperature [47,53,54]:

σ = Σ n_i_·q_i_·µ_i_ (8)

where n_i_, q_i_ and μ_i_ are the charge carrier density, 1.6 × 10^−19^ C, and ion mobility, respectively. The increase in both charge carrier concentration (n) or in the ionic species mobility in the system cause an increase in ionic conductivity (s), as Equation (10) clearly verified.

**Figure 5:**
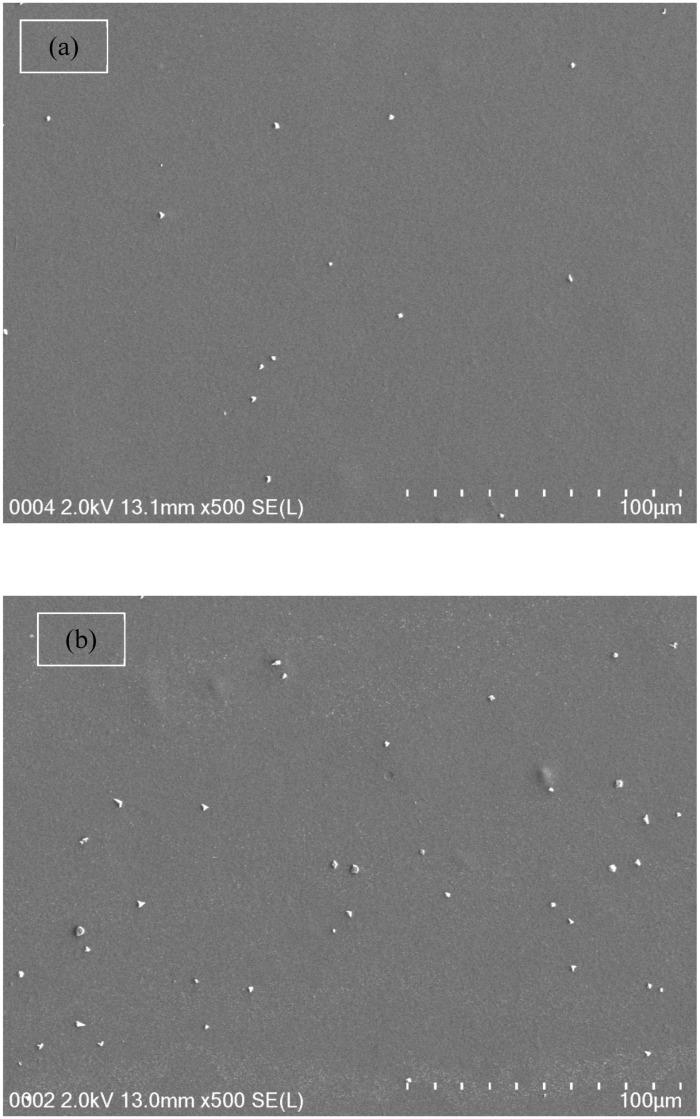

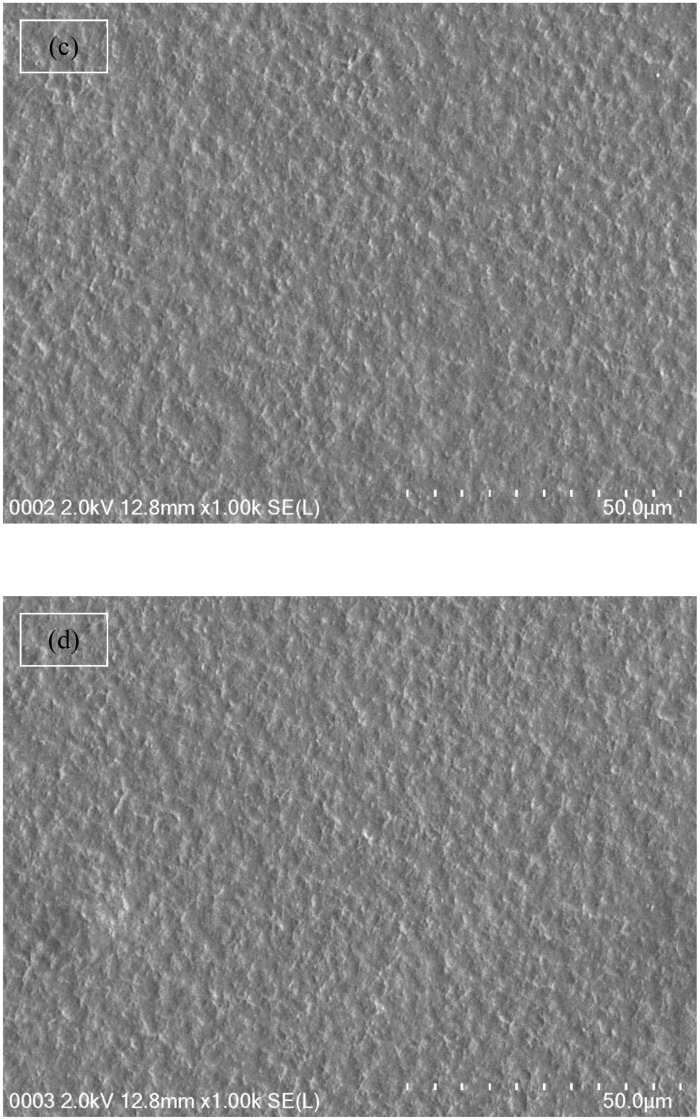
Field emission scanning electron microscopy (FESEM) images for (**a**) CSDX 1, (**b**) CSDX 2, (**c**) CSDX 3, and (**d**) CSDX 4 blend electrolytes.

From both Table 1 and impedance plots, it can be clearly observed that the DC conductivity increases with an increase in ammonium fluoride salt concentration. It is well documented that in proton conducting solid polymer based electrolytes, a channel for proton transport builds up and can clearly be seen as white specs on the film surfaces (see Figure 5a–d) [55]. It is obvious from Figure 5a,b that the appearance of a number of white specs increases with an increase in ammonium fluoride salt from 10 to 20 wt.% whereas from 30 up to 40 wt.% resulted in a uniform complex formation with the presence of many dark region holes. This complex is believed to be the ion traps/particles model in the polymer blend host that is responsible for the conduction process [56]. Another observation is the surface that has become denser with a massive number of dark holes. These holes can be considered as spaces or hosts for salt to fill, which in turn resulted in ionic conduction [57]. As a consequence, this change in the morphology of the surface indicates a lowering of the degree of crystallinity [58]. For example, at high salt concentrations, the FESEM image reveals the maximum dominancy of the dark region, indicating the high amorphous phase within the blend electrolyte and as confirmed in impedance plots.

### 3.3. EDLC Study

#### 3.3.1. Transference Number Measurement (TNM) Study

**Figure 6:**
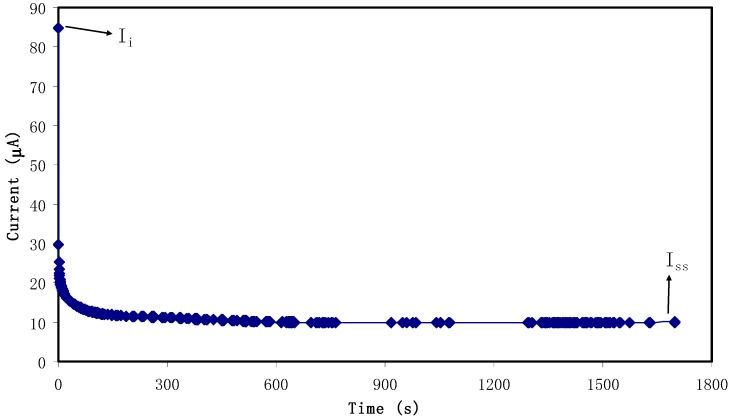
Polarization current versus time for the highest conducting (CSDX4) electrolyte film.

Transference number measurement (TNM) verified the main or dominant charge carrier species in the polymer electrolyte. Figure 6 shows the polarization curve where at 0.80 V, current is recorded until it showed saturation for the relatively highest conducting electrolyte. Accordingly, current decayed drastically before reaching the steady state. During steady state, the polarization of the cell occurred and the remaining current flow was due to electrons rather than ions. This is due to blocking caused by ions at the stainless steel electrodes where only electrons can pass through [59]. The values of *t_i_* and *t_e_* were calculated using Equations (1) and (2) and the values of *I_i_* and *I_ss_* were extracted as 84.9 μA and 10 μA, respectively and *t_i_* value is 0.88. This result is in good accordance with that of the carboxylmethylcellulose-NH_4_F system reported by Ramlli & Isa [60]. As a result, in the chitosan-dextran-NH_4_F system, ions are found to be the main charge carriers during the migration process.

#### 3.3.2. Electrochemical Stability Determination

**Figure 7:**
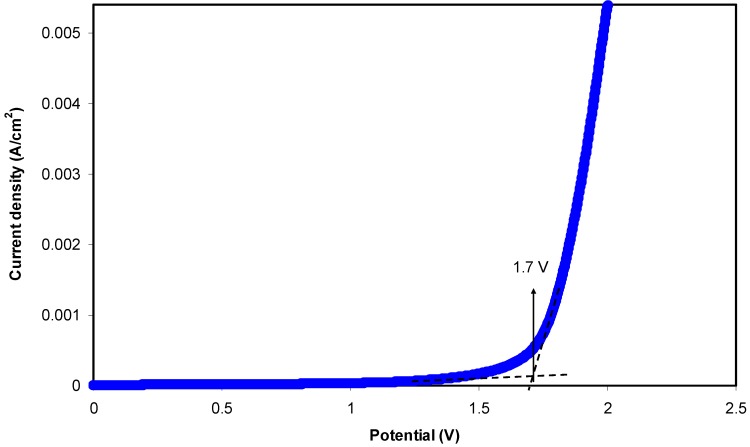
Linear sweep voltammetry (LSV) plot for the highest conducting (CSDX4) blend electrolyte film.

**Figure 8:**
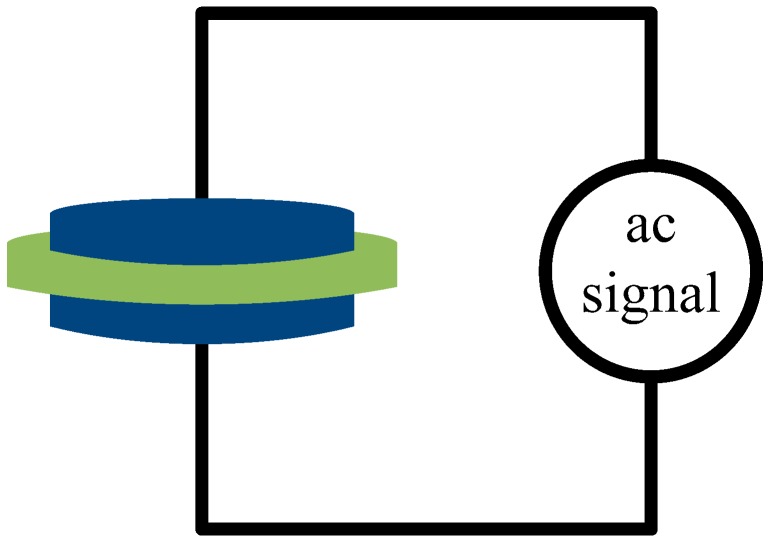
Schematic diagram for electrochemical stability measurement.

The study of electrochemical stability is only carried out for the highest conducting chitosan-dextran-NH_4_F system using linear sweep voltammetry (LSV). Figure 7 shows the LSV plot for the sample. The voltage was swept from 0 V to 3 V until a relatively large current is observed at a certain potential at a scan rate of 50 mV s^−1^. At a low potential, no obvious current is observed until 1.70 V. This large current beyond 1.70 V resulted from the decomposition of electrolyte at the inert electrode surface. This result also signifies that neither reduction nor oxidation is experienced by the chitosan-dextran-NH_4_F system in 0 to 1.70 V [61]. It is evident that there is no current flowing beneath 1.70 V, which points out that there is no electrochemical reaction occurring below this potential window. Thereby, the chitosan-dextran-NH_4_F system is found to be steady up to 1.70 V, which is still appropriate for applications in proton based energy devices. This result showed comparability with other ammonium salt based polymer electrolytes. Similarly, a plasticized system of chitosan-polyvinyl alcohol-NH_4_NO_3_ with electrochemical stability up to 1.70 V was documented by Kadir and Arof [62]. Noor and Isa [63] also explored the cellulose-NH_4_SCN system being electrochemically durable up to 1.70 V. The schematic diagram for electrochemical stability measurement is shown in Figure 8.

#### 3.3.3. Cyclic Voltammetry Test for the EDLC

**Figure 9:**
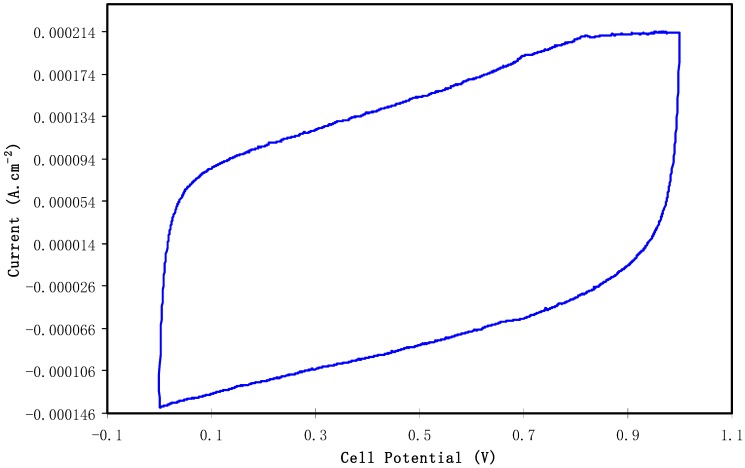
Cyclic voltammetry (CV) plot of the fabricated electrical double layer capacitor (EDLC) from CSDX4 film in the potential range of 0 V to 1 V.

**Figure 10:**
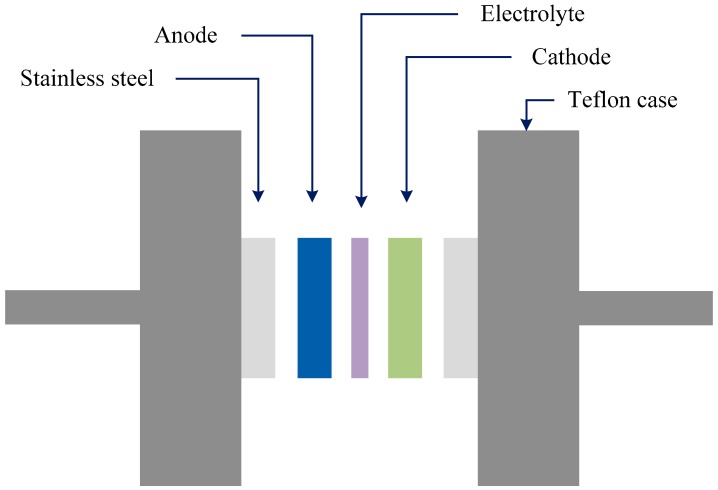
Schematic configuration for EDLC cell.

The characterization of the double-layer of the fabricated EDLC was performed with cyclic voltammetry (CV) at 100 mV s^−1^ as presented in Figure 9. The CV profile of the fabricated EDLC is almost a rectangular shape. Shuhaimi et al. [64] stated that a perfect rectangular shape is the shape of a perfect capacitor with an absence of a peak where non-Faradaic reactions proceed. From the current results, one can notice the rectangular shaped CV curve as evidence of a rapid current response to the applied voltage. This pattern of the CV curve is quite comparable to other EDLC systems that are documented in the literature [65,66,67]. A more interesting observation is the absence of oxidation and reduction peaks in the CV curve portraying that no intercalation/deintercalation occurs. This signifies that the energy storage mechanism of the EDLC is via ion adsorption and the accumulation of charge double-layer at the interface of the activated carbon. In other words, cations from the electrolyte and electrons from the electrodes form potential energy [68]. It is well established that the conceptual principle of capacitance comes from non-Faradaic (double layer capacitor)/or Faradaic (pseudocapacitor) charge transfer. If clear redox-peaks are encountered, then the behavior contains battery-like components up to the point where only insignificant capacitor-like contributions to the charge storage capacity are found and the concept of capacitance becomes unsuitable [69]. From the above discussion it is understood that the leaf-like shape is evidence of the fact that the characteristics of the prepared EDLC are close enough to ideal rectangular shape capacitors. In other words, the non-existence of battery-like components in the CV plot confirms the non-Faradaic process in the EDLC device of the present work. The schematic configuration for the EDLC cell is shown in Figure 10.

#### 3.3.4. EDLC Characteristics

**Figure 11:**
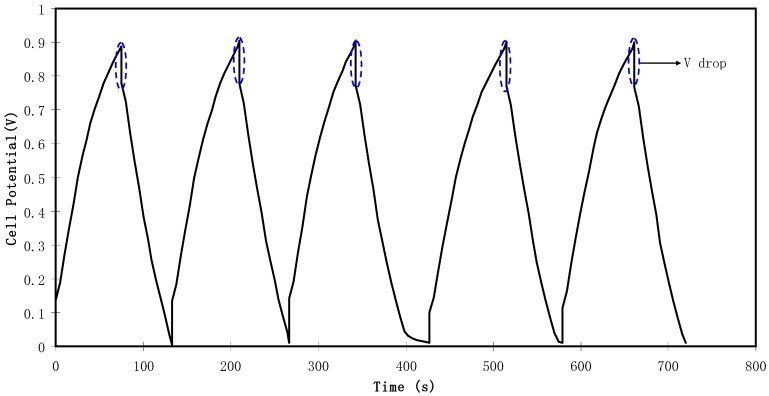
Charge-discharge profiles for the fabricated EDLC at 0.5 mA cm^−2^.

**Figure 12:**
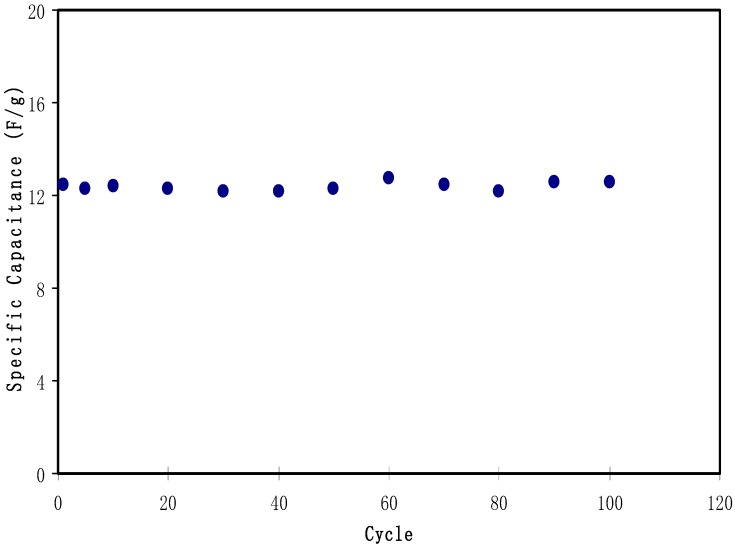
Specific capacitance of the fabricated EDLC for 100 cycles.

Figure 11 exhibits the typical galvanostatic charge-discharge characteristics of the fabricated EDLC. Other evidence of the existence of charged double-layer or capacitive characteristics is further verified via the gradient of the discharge parts which is almost linear [70]. The values of *C_sp_* of the EDLC were calculated and one can extract the value of the gradient of each discharge part from the plot using Equation (3). Figure 12 illustrates the *C_sp_* value of the EDLC up to 100 cycles. It is noticeable that *C_sp_* is almost constant from 1st cycle to 100th cycle with an average of 12.4 Farad/g. The achieved value of specific capacitance in the current study is of great interest compared to those reported for other polymer based electrolytes. For example, 4 Farad g^−1^, 4.3 Farad g^−1^, 8.4 Farad g^−1^ and 6.5-15 Farad g^−1^ were obtained for PEO9/LiCF_3_SO_3_ plasticized with 50 wt.% PEG200 (PEO–NAPP)11/LiClO_4_, PVDF–HFD(25%)+ PC10–EC10/LiClO_4_Nafion 1100 ionomer swelled membranes, Polyurethane8/LiClO_4_ and PVA–cellulose–H_3_PO_4_ based electrolytes, respectively [71].

**Figure 13:**
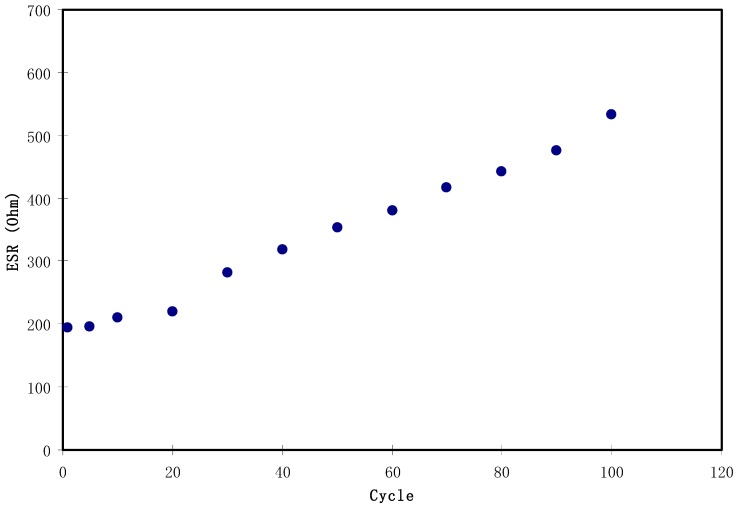
The pattern of equivalent series resistance of the EDLC for 100 cycles.

Figure 11 shows a potential drop before the start of every discharging process. This potential drop is owing to the presence of internal resistance called equivalent series resistance (*R_es_*) which was determined using Equation (4). As depicted in Figure 13, the *R_es_* value of the EDLC is in the range of 194.5 to 533 Ω. The presence of *R_es_* in the EDLC is mainly due to three resistive parts, for example, the current collector (aluminum foil), the bulk of the electrolyte as well as the interfacial region between electrolyte and electrodes [72].

**Figure 14:**
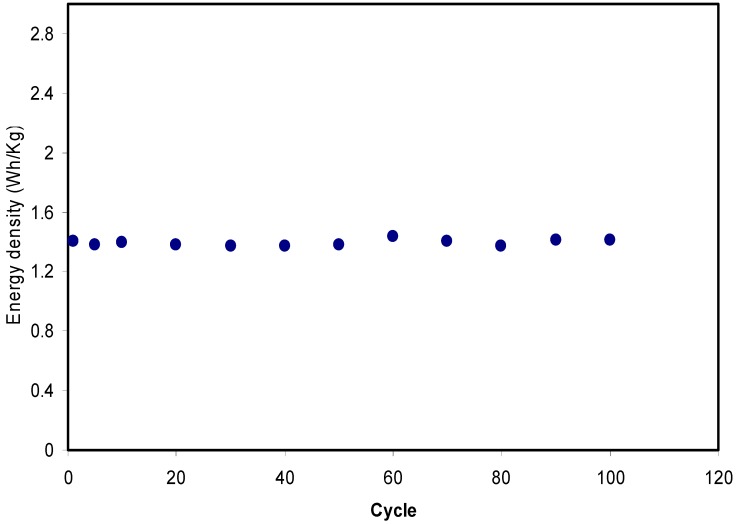
Energy density of the fabricated EDLC for 100 cycles.

**Figure 15:**
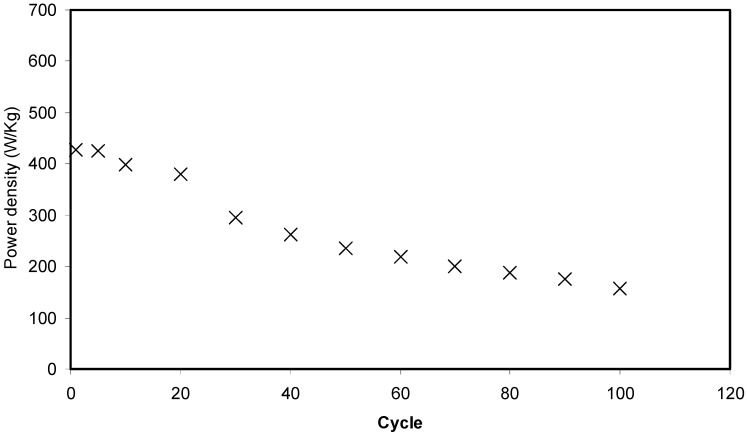
Power density of the fabricated EDLC for 100 cycles.

Figure 14 shows that the pattern of *E* is the similar to *C_sp_* where it remains constant from the 1st cycle to 100th cycle. The average value of *E* for exactly 100 cycles is found to be 1.40 Wh kg^−1^. Herein, ions are taken into consideration that were involved in the conduction process with a comparable energy barrier throughout the charge and discharge process [73]. Hamsan et al. [24] recorded the same energy density (*E*) constant at 2.2 Wh kg^−1^ for the methycellulose-starch-NH_4_NO_3_ EDLC system. This high value of *E* may belong to a lower lattice energy of NH_4_NO_3_ (648.9 kJ mol^−1^) than NH_4_F (834.0 kJ mol^−1^) [65,74]. It is self-evident; low lattice energy means an easy dissociation process, therefore providing too many ions for polarization. In the current work, the obtained energy density (1.40 Wh kg^−1^) of the EDLC cell is of great interest compared to that recorded (0.3 Wh/Kg) for the Ionic liquid incorporated PEO based polymer electrolyte [75]. Figure 15 exhibits the plot of *P* versus the cycle number, which is obtained from Equation (6). The value of *P* is 428.4 W kg^−1^ at the 1st cycle where it experiences a slight drop at the 2nd cycle. The value of *P* decreased as the cycle number increased. This reduction trend harmonized with an increase trend of *R_es_* as shown in Figure 13. The increment of internal resistance is correlated with the depletions of the electrolyte and the recombination of ions regarding the fast charge and discharge mechanism; as a result, it delivers lower power density at a high cycle number [76]. Energy density is how much energy an EDLC can store while the power density is how much power an EDLC can deliver. Hence, the increment in *R_es_* value does not affect the trend of energy density. The trend of *C_sp_*, *R_es_*, *E* and *P* for the fabricated EDLC in this work is similar to that found in other EDLC studies [3,67]. The authors stated that *R_es_* is related to the power density of the EDLC.

## 4. Conclusions

In conclusion, polymer blend electrolytes are of a great interest for EDLC applications. The structural analysis exhibited broad amorphous peaks in the XRD spectra as a result of the disruption of hydrogen bonding within the polymer chains. The formation evident technique indicated the complex formation after the addition of ammonium salt and functional groups via a shift and a decrease in the intensity of FTIR bands. Using various concentrations of NH_4_F in the CS:Dextran blended electrolyte, the highest conductivity of 1 × 10^−3^ S/cm was obtained with 40 wt.%.The FESEM image supports the characterization of the conductivity behavior of the samples through the appearance of channels for ion transport and many dark regions belonging to the amorphous phase. Transference number measurement (TNM) of the highest conducting electrolyte was obtained at 0.88 which reveals the dominancy of ions in the conduction process. Linear sweep voltammetry (LSV) verifies that the blend electrolyte can be used in electrochemical devices with electrochemical stability up to 1.7 V. Based on TNM, LSV and conductivity results, the fabrication of the electrical double layer capacitor (EDLC) was carried out. The performance of the EDLC cell has been studied by cyclic voltammetry and constant current charge–discharge techniques at ambient temperature. The nearly rectangular shape for the CV plot was observed with no redox peaks. The stability of the fabricated EDLC over 100 cycles is another achievement as well as the relatively low equivalent circuit resistance and high power density at the 1st cycle. Both the specific capacitance and energy densities are almost constant over 100 cycles.

## Figures and Tables

**Table 1:** Conductivity for pure CS:Dex and blend electrolyte films at room temperature.

Sample Designation	DC Conductivity (S/cm)
CS:Dex	1.2 × 10^−10^
CSDX1	5.9 × 10^−8^
CSDX2	2.2 × 10^−7^
CSDX3	1.7 × 10^−4^
CSDX4	1 × 10^−3^
